# How to measure and model cardiovascular aging

**DOI:** 10.1093/cvr/cvaf138

**Published:** 2025-08-28

**Authors:** Luke Spray, Gavin Richardson, Laura K Booth, Judith Haendeler, Joachim Altschmied, Daniel I Bromage, Sienna B Wallis, Konstantinos Stellos, Simon Tual-Chalot, Ioakim Spyridopoulos

**Affiliations:** Translational and Clinical Research Institute, Vascular Biology and Medicine Theme, Faculty of Medical Sciences, Newcastle University, Centre for Life, Newcastle Upon Tyne NE1 3BZ, UK; Cardiology Department, Freeman Hospital, Newcastle upon Tyne NE7 7DN, UK; Biosciences Institute, Vascular Biology and Medicine Theme, Faculty of Medical Sciences, Newcastle University, Centre for Life, Newcastle Upon Tyne NE1 3BZ, UK; Biosciences Institute, Vascular Biology and Medicine Theme, Faculty of Medical Sciences, Newcastle University, Centre for Life, Newcastle Upon Tyne NE1 3BZ, UK; Cardiovascular Degeneration, Clinical Chemistry and Laboratory Diagnostics, Medical Faculty, University Hospital and Heinrich-Heine University Duesseldorf, Duesseldorf 40225, Germany; CARID, Cardiovascular Research Institute Düsseldorf, Medical Faculty, University Hospital and Heinrich-Heine University Duesseldorf, Duesseldorf 40225, Germany; Cardiovascular Degeneration, Clinical Chemistry and Laboratory Diagnostics, Medical Faculty, University Hospital and Heinrich-Heine University Duesseldorf, Duesseldorf 40225, Germany; CARID, Cardiovascular Research Institute Düsseldorf, Medical Faculty, University Hospital and Heinrich-Heine University Duesseldorf, Duesseldorf 40225, Germany; King’s College London British Heart Foundation Centre of Excellence, School of Cardiovascular Medicine and Sciences, London, UK; Translational and Clinical Research Institute, Vascular Biology and Medicine Theme, Faculty of Medical Sciences, Newcastle University, Centre for Life, Newcastle Upon Tyne NE1 3BZ, UK; Biosciences Institute, Vascular Biology and Medicine Theme, Faculty of Medical Sciences, Newcastle University, Centre for Life, Newcastle Upon Tyne NE1 3BZ, UK; Department of Cardiovascular Research, European Center for Angioscience (ECAS), Medical Faculty Mannheim, Heidelberg University, Mannheim, Germany; German Centre for Cardiovascular Research (DZHK), Partner Site Heidelberg/Mannheim, Mannheim, Germany; Helmholtz-Institute for Translational AngioCardioScience (HI-TAC) of the Max Delbrück Center for Molecular Medicine in the Helmholtz Association (MDC) at the Heidelberg University, Heidelberg, Germany; Department of Medicine, University Medical Centre Mannheim, Heidelberg University, Mannheim, Germany; Biosciences Institute, Vascular Biology and Medicine Theme, Faculty of Medical Sciences, Newcastle University, Centre for Life, Newcastle Upon Tyne NE1 3BZ, UK; Translational and Clinical Research Institute, Vascular Biology and Medicine Theme, Faculty of Medical Sciences, Newcastle University, Centre for Life, Newcastle Upon Tyne NE1 3BZ, UK; Cardiology Department, Freeman Hospital, Newcastle upon Tyne NE7 7DN, UK

**Keywords:** Aging, Inflammation, Senesence, Cardiovascular disease, Modelling

## Abstract

Most acquired cardiovascular diseases are more common in older people, and the biological mechanisms and manifestations of aging provide insight into cardiovascular pathophysiology. Measuring aging within the cardiovascular system may help to better understand risk profiles for specific individuals and direct targeted preventative therapy. In this review, we explore telomere attrition, cellular senescence, epigenetic modifications, and mitochondrial dysfunction as key molecular mechanisms of aging. These phenomena are associated with cardiovascular disease through endothelial dysfunction and systemic inflammation, which are measurable in clinical practice with a variety of clinical, laboratory, and imaging techniques. Finally, we discuss that the next tools for modelling cardiovascular aging must be capable of incorporating a vast amount of diverse data from a given patient, pointing to recent developments in artificial intelligence and machine learning.

**This article is part of the Spotlight Issue on Ageing.**

## 1. Introduction

Cardiovascular aging is a fundamental process that contributes to frailty and the development of various life-threatening diseases, including hypertension, heart failure (HF), aortic valve disease, and atherosclerosis.^1–3^ As life expectancy continues to rise globally, the burden of age-related cardiovascular conditions has become a critical determinant of both healthspan—the period of life spent in good health—and overall lifespan. The increasing prevalence of these conditions highlights the urgent need to understand and mitigate the effects of cardiovascular aging.

The cardiovascular system plays a multifaceted role in maintaining physiological homeostasis. Beyond its primary function of delivering oxygen and essential nutrients to all tissues while removing metabolic waste, it serves as a central regulator of interorgan communication. The intricate network of blood vessels not only supplies vital resources but also facilitates the exchange of biochemical signals between organs, influencing metabolic, immune, and neurological functions. This complex interplay underscores the circulatory system’s role as a gatekeeper of healthy aging.

With advancing age, vascular dysfunction, endothelial impairment, and arterial stiffening progressively disrupt cardiovascular homeostasis.^4,5^ These changes increase the risk of systemic inflammation, oxidative stress, and impaired tissue perfusion, all of which accelerate biological aging processes. Measuring and modelling cardiovascular aging have therefore become crucial for identifying early markers of decline and developing targeted interventions. In this review, we focus on imaging, biomarker analysis, and computational modelling that could pave the way for personalized strategies to delay or even reverse cardiovascular aging, ultimately promoting longevity and quality of life.

## 2. Biological basis of cardiovascular aging

### 2.1 Telomeres and telomerase

Telomeres, located at the ends of chromosomes, consist of tandem repeats of the hexanucleotide sequence TTAGGG and form a higher-order structure stabilized by DNA-binding proteins.^6^ These structures prevent degradation, recombination, and chromosome fusions as well as the recognition of chromosome ends as DNA double-strand breaks. Telomere attrition, the gradual shortening of telomeres with each cell division, is a fundamental process of aging. It is an inevitable process, which occurs due to the so-called end replication problem, describing the circumstance that the replication machinery cannot fully duplicate the end of linear double-stranded DNA.^7^ After a critical limit of cell divisions, this process leads to cellular senescence, a hallmark of aging.^8,9^ This telomere erosion is counteracted by the enzyme telomerase, which—as holoenzyme—consists of the catalytic subunit Telomerase Reverse Transcriptase (TERT), the Telomerase RNA Component (TERC), and several accessory proteins.^10^

#### 2.1.1 Telomere length in atherosclerosis and HF

The most easily accessible patient material is blood, which allows the determination of telomere length in circulating cells by various methods.^11^ In humans, shortened telomeres in leukocytes have been consistently observed in individuals with coronary artery disease (CAD) and HF.^12–14^ Our own studies have shown that telomere shortening occurs uniformly across various haematopoietic compartments, including bone marrow–derived myeloid cells and thymic progenitor cells, suggesting that leukocyte telomere length (LTL) attrition reflects systemic influences such as increased cellular turnover or DNA damage due to inflammation and oxidative stress—common features of aging—rather than being a direct cause of chronic disease .^15,16^

A large-scale meta-analysis involving 14 studies and over 200 000 participants reported a linear inverse association between LTL and CAD risk: each 1 kb increase in telomere length was associated with an approximate 23% reduction in coronary heart disease (CHD) risk.^12^ These findings support LTL as a robust biomarker of atherosclerotic cardiovascular risk.

While the association between short LTL and CHD is well established, evidence for a causal relationship remains inconclusive. Mendelian randomization (MR) offers a powerful strategy to address issues of confounding and reverse causation by using genetic variants as proxies for lifelong exposure.^17^ A two-sample MR study of over 470 000 individuals demonstrated that genetic determinants of longer telomeres were associated with a modest but statistically significant reduction in CAD risk within European-ancestry populations, though no causal relationship was observed for cerebral or peripheral atherosclerosis.^18^

Scheller Madrid *et al*.^19^ further explored this question using MR in a cohort of 290 000 individuals. They examined three single nucleotide polymorphisms (SNPs) associated with reduced telomere length—located in TERT, TERC, and OBFC1. They found modest but statistically significant increases in ischaemic heart disease risk for variants in TERT (RR: 1.04; 95% CI: 1.02–1.06) and OBFC1 (RR: 1.05; 95% CI: 1.03–1.08), whereas the TERC variant, although associated with shorter telomeres, showed no significant association with disease risk (RR: 1.01; 95% CI: 0.99–1.03). Unlike the protein-coding TERT and OBFC1, TERC encodes the RNA template essential for telomerase activity. These findings raise the possibility that telomere length may serve as a downstream marker of diminished telomerase function, rather than being intrinsically pathogenic. Thus, shorter telomeres alone may not be sufficient to drive pro-inflammatory diseases such as atherosclerosis.

In a UK Biobank cohort of 40 459 middle-aged adults, longer LTL was associated with favourable cardiac remodelling parameters on cardiac MRI and a reduced incidence of HF over a median follow-up of 12 years (HR: 0.86 for highest vs. lowest quartile).^13^ Similarly, a larger UK Biobank analysis of ∼403 000 individuals without pre-existing cardiovascular disease (CVD) found that individuals in the lowest LTL quartile had a significantly higher incidence of sudden cardiac death, coronary events, and HF hospitalizations.^20^ Another UK biobank study using MR in over 470 000 participants suggested that shorter telomere length can decrease life span up to 2.5 years.^21^ These studies suggest that longer telomeres may confer resilience against structural cardiac decline and support the concept of LTL as a biomarker of biological rather than chronological aging.

At the myocardial level, cardiomyocytes from patients with HF exhibit significantly shorter telomeres than those from healthy controls. Functional studies using patient-derived induced pluripotent stem cell cardiomyocytes revealed that telomere shortening leads to chromatin remodelling and upregulation of the developmental transcription factor Forkhead Box C1. This, in turn, promotes cellular senescence and contractile dysfunction.^22^ These findings provide a mechanistic basis for the clinical observation that short telomeres are associated with poor cardiac outcomes, establishing a direct link between telomere attrition and myocardial failure.

#### 2.1.2 Telomerase and mitochondrial function

Telomere maintenance is undoubtedly critical in stem cells, germ cells, and tissues with high proliferative capacity. However, other aging-related mechanisms could be more important in slowly or non-dividing cells, like neurons and the major structural cell types of the cardiovascular system, cardiomyocytes, endothelial cells (ECs), vascular smooth muscle cells, and fibroblasts. Here, mitochondrial dysfunction, another hallmark of aging,^9^ might be more relevant. The functions of mitochondria reach far beyond energy provision as they integrate multiple metabolic signals and, thus, serve as a central node in metabolism^23^ and signalling organelles.^24,25^ Moreover, they are at the center of oxidative metabolism, as they detoxify molecular oxygen in the respiratory chain and themselves are one of the major intracellular producers of reactive oxygen species (ROS).^26^ Thus, they play an important role in the cellular redox homeostasis, which becomes disturbed with increasing age resulting in oxidative stress.^27,28^ The constant work of cardiomyocytes consumes enormous amounts of adenosine triphosphate (ATP), generated by mitochondria, but ECs, vascular smooth muscle cells, and fibroblasts all also rely on proper mitochondrial function.^29–31^ Mitochondrial dysfunction is therefore a typical feature of CVD.^32^

Interestingly, TERT is also critical for normal mitochondrial function. The holoenzyme telomerase was originally identified as a nuclear enzyme responsible for telomere maintenance in the unicellular eukaryote *Tetrahymena*^33^ and subsequently also in humans.^34^ Nearly two decades later, its catalytic subunit TERT has been detected in mitochondria of human cells by several independent groups.^35–38^ Localization of TERT in these organelles can be explained by the presence of a *bona fide* mitochondrial targeting sequence at the N-terminus of the mammalian protein,^35,37^ in addition to the nuclear import and export signals,^39,40^ allowing transport of TERT into either of the two organelles. However, the mode of action in mitochondria must be different from the nucleus as the circular mitochondrial DNA (mtDNA) does not contain telomeres and because TERC is not imported into mitochondria.^41^ A direct link between TERT and mitochondrial functions was originally provided by the observations that TERT binds to mtDNA and protects it against damage,^37,41^ reduces mitochondrial superoxide levels^36,37,40,41^ and is required for full complex I activity,^37,42^ several processes interrelated to each other. Moreover, expression of a TERT mutant defective in mitochondrial import led to ultrastructural changes in mitochondria,^41^ while expression of TERT forced into the mitochondria restored nitric oxide (NO)-mediated dilation of microvessels from patients with CAD.^43^

All studies to this point were either performed by expressing TERT forced into the cell nucleus or the mitochondria by disruption or addition of specific targeting signals, on a TERT-proficient cellular background, or in global TERT-knockout animals and organs thereof. This made it difficult to unequivocally assign specific effects to nuclear or mitochondrial TERT, respectively, especially *in vivo*. This dilemma was solved by the generation of two unique mouse models containing TERT exclusively in one of the cellular compartments in all cells of the body.^44^ Using these mice, it was shown that mitochondrial, but not nuclear TERT is necessary and sufficient to maintain mitochondrial complex I activity and to ameliorate ischaemia/reperfusion injury of the heart. The latter can be ascribed—at least in part—to protection of cardiomyocytes against apoptosis, improved revascularization, and enhanced myofibroblast differentiation.^44^

Thus, mitochondrial TERT has a protective effect with respect to age-related CVDs, and it may be desirable to increase the levels of TERT in mitochondria in humans. Such an effect has been demonstrated with TA-65®, a purified extract from the medicinal plant *Astragalus membranaceous* in primary human ECs, along with improved migratory capacity^44^ and reduced ROS release in brain mitochondria.^45^ Moreover, dietary restriction increases mitochondrial TERT levels in the brain.^46^ In a clinical setting, remote ischaemic preconditioning in patients undergoing coronary artery bypass grafting, which had a cardioprotective effect that was accompanied by improved mitochondrial respiration,^47^ also led to an increase in mitochondrial TERT levels in right atrial appendages.^44^

**Figure 1:**
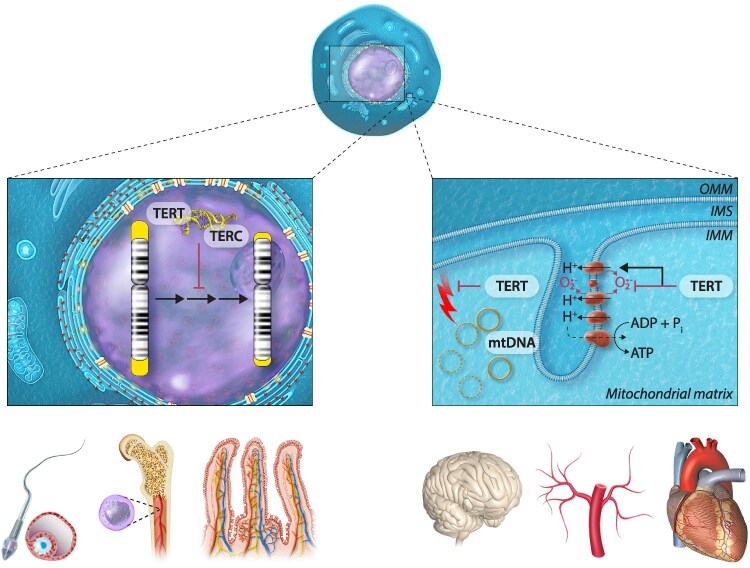
Telomerase reverse transcriptase counteracts different hallmarks of aging through dissimilar, organelle-specific mechanisms. In the nuclei of germline cells, stem cells, e.g. in the bone marrow or in intestinal crypts and of rapidly dividing cells like the intestinal epithelium, the telomerase holoenzyme consisting of TERT, TERC, and accessory proteins prevents telomere erosion and, thus, induction of cellular senescence. Conversely, in slowly or non-dividing cells of e.g. the brain, the vasculature and the heart, mitochondrial TERT protects mitochondrial DNA against damage, maintains activity of respiratory chain complex I and limits superoxide production, thereby maintaining mitochondrial functionality. TERT, telomerase reverse transcriptase; TERC, telomerase RNA component; OMM, outer mitochondrial membrane; IMS, intermembrane space; IMM, inner mitochondrial membrane; mtDNA, mitochondrial DNA.

In summary, TERT uses different modalities to counteract at least two hallmarks of aging, namely telomere erosion and mitochondrial dysfunction (*Figure 1*). In rapidly dividing cells, its most important role might be the prevention of telomere erosion. Its predominant effect in cells with low proliferative capacity, such as those in the brain and the cardiovascular system, appears to be the maintenance of mitochondrial function, specifically supporting electron transport chain function and restricting production of ROS.

### 2.2 Senescence

On a whole-organism level, a recognized feature of aging pathologies is the accumulation of senescent cells: these are defined as cells, which have irreversibly exited the cell cycle and display a particular combination of characteristics including overexpression of pro-survival Bcl-2 family proteins and expression of the pro-inflammatory senescence-associated secretory phenotype (SASP).^48,49^ From its initial definition as simply replicative exhaustion,^50^ it is now known that cellular senescence is a complex phenotype, which can be acquired in a telomere-independent fashion by post-mitotic cell types, including cardiomyocytes, and can contribute to disease in various organ systems, including atherosclerosis,^51,52^ myocardial infarction (MI),^53^ and HF.^54,55^ In addition to replicative exhaustion, senescence can be induced by a range of stressful stimuli, termed stress-induced premature senescence, with the common features of oxidative stress and mitochondrial dysfunction. Overproduction of ROS, or inadequate antioxidant processes, leads to DNA damage and a DNA damage response (DDR).^56^ DDR then leads to cell cycle arrest, through activation of the p53/p21 pathway, and senescence.

Multiple cardiovascular risk factors beyond age are also closely linked with senescence. Hyperglycaemic media shifts cells towards senescence *in vitro*, and senescence of pancreatic islet cells has been implicated in the development of diabetes.^57^ High levels of lipids also contribute to senescence as, in the presence of ROS, lipids are oxidized and cause ER-stress.^58^ White adipose tissue (WAT) is especially prone to senescence, and this is accelerated in the presence of obesity or diabetes.^59,60^ In models of obesity, senescent WAT cells appear very early in the disease process, suggesting senescent cells may contribute to the core pathogenesis of obesity and insulin resistance.^61–63^ Cigarette smoking also accelerates senescence,^64^ and in mouse models targeting senescence improves smoking-related lung disease.^64^ Sarcopenia, the loss of muscle mass which commonly occurs with aging, is also strongly linked to senescence and CVD incidence.^65^

Attenuated macroautophagy and an accumulation of dysfunctional mitochondria via impaired mitophagy are recognized hallmarks of aging,^9^ and of cellular senescence.^48,66^ Accordingly, short hairpin RNA–based knockdown of key mitophagy components, such as PINK1, Parkin, or p62, is sufficient to induce senescence. Further, several pharmacological interventions identified as ‘anti-senescent’ have been found to promote mitophagy, with their anti-senescent effects being dependent on functional expression of the autophagic machinery.^67^ In the context of CVD and myocardial aging, mouse models have demonstrated that functional autopaphy within cardiomyocytes is critical to maintain myocardial health. Disruption in autophagy via cardiomyocyte-specific deletion of Atg5 results in myocardial dysfunction with characteristics of age-related remodelling.^68^ In non-cardiomyocyte cell lineages, including fibroblasts and primary human cardiac microvascular ECs, enhancing autophagy has been shown to have therapeutic potential. Novel small molecules that activate mitophagy machinery, such as p62, have shown promising anti-senescence and anti-aging properties.^67^ The action of many longevity-promoting interventions often converges on the activation of AMP kinase (AMPK), activation of the Sirtuin pathway, or dampening of mechanistic target of rapamycin. Some suggest that widespread changes in these complex and far-reaching pathways (such as by administering rapamycin) are regulated in the cardiac microenvironment by post-transcriptional mechanisms such as micro RNA (miRNA) networks.^69,70^ Certainly, more precise molecular mechanisms and targeting strategies must be elucidated to maximize the potential of any longevity-promoting intervention. Although tracking changes in mitophagic flux in real time is technically challenging, fluorescent reporters have recently been used both *in vitro* alongside orthogonal studies, and *in vivo*, to great effect.^67,71^

The mechanisms by which enhanced autophagy appears to improve cellular function centre on improved mitochondrial function, as a result of increased mitophapy and mitochondrial turnover. This is perhaps unsurprising, as dysfunctional mitochondria contribute to several pathological processes related to aging. Mitochondria act as a source of oxidants in the cell, and mitochondrial dysfunction (e.g. decreased membrane potential and increased proton leak) accelerates senescence in large part by release of ROS.^72,73^ Mitochondria-derived ROS form part of the SASP and studies using mitochondria-depleted senescent cells show that the organelles are in fact required for expression of the SASP; mitochondria-depleted senescent cells remain in cell cycle arrest but fail to express the classical senescence markers p16 and p21, or the typical pro-inflammatory and pro-oxidant phenotype.^74^ It has been suggested that increased permeability of the outer mitochondrial membrane in senescent cells allows the release of mtDNA, which can activate the cGAS-STING pathway (normally responsible for recognising exogenous, pathogenic, cytoplasmic DNA).^75,76^ Additionally, against the backdrop of increased pro-survival pathway expression, insufficient cytochrome c release and caspase activation in a senescent cell may result in sublethal apoptotic processes, which induce DNA damage and contribute to further genetic instability.

Some argue that nuclear DNA leakage may also be a trigger for cellular DNA sensors and inflammation. Senescent cells have been shown to extrude chromatin fragments from their nuclei to the cytoplasm, thereby triggering the cGAS-STING pathway.^77^ Consequently, NF-kB signalling is activated, transcription of pro-inflammatory genes is switched on, and SASP released. Other DNA sensors may also be activated by nuclear or mtDNA in senescent cell cytoplasm, including toll-like receptor 9, and the inflammasome complex. Clinically, there is significant interest in how circulating mtDNA correlates with hypertension,^78^ and how cell-free mtDNA is associated with MI.^79^ Dampening DNA-leakage sensors using small molecules has been investigated in preclinical studies,^80^ and further research is warranted to assess their potential in senescence and CVD.

With aging being the biggest risk factor for CVD, prophylactically or curatively abrogating senescent cell burden in the aging heart is an exciting concept. Landmark studies explored this notion using senolytics: pharmacological agents, such as Navitoclax, which selectively induce senescent cells to apoptosis by inhibiting Bcl-2 family proteins.

Suggesting that senescent cells may be detrimental post-MI, the use of Navitoclax in mice post-MI has been shown to attenuate cardiomyocyte hypertrophy and myocardial profibrotic TGFβ2 expression.^81^ Standard treatment of MI involves reperfusion of the ischaemic area of myocardium, but this sudden reperfusion is itself associated with localised oxidative stress and inflammation, termed ischaemia-reperfusion injury (IRI).^82^ In an IRI setting, Navitoclax treatment was associated with reduced infarct scar size, increased angiogenesis, and reduced SASP expression.^53,81^

Though the heart is thought to have limited regenerative capacity overall, a small population of cardiac progenitor (or stem) cells (CPCs) are thought to underpin this capacity, which is important for reparative potential post-insult. In patients > 70 years of age, over half of CPCs have been shown to be senescent, and unable to fulfil their regenerative, reparative role in an infarcted heart. Furthermore, the SASP of these CPCs was able to induce senescence in non-senescent CPC populations *in vitro*, but the addition of senolytic combination therapy with dasatinib and quercetin abrogated these effects.^83^

Overall, several aspects of the senescent cell phenotype may lend themselves to promotion of CVD in an aging setting, including the pro-inflammatory SASP and the loss of any limited regenerative potential. The prospect of senolytics is exciting, but the long-term effects of removing cells from a post-mitotic cardiomyocyte population are uncertain. It is heartening, however, that Navitoclax is being employed in Phase II clinical trials in an oncology setting and shows a favourable safety profile. Novel approaches such as senomorphics (drugs which modify or dampen the senescent phenotype, particularly SASP) may hold more promise, but even these show mixed efficacy from preclinical studies.^84^ Certainly, a better understanding of the detrimental aspects of the senescent phenotype will allow for more targeted therapeutic approaches.

### 2.3 Epigenetics

One of the key mechanisms contributing to the chronic low-grade inflammation observed in aging cardiovascular systems is driven by dynamic and flexible age-related epigenetic modifications. These modifications, including DNA methylation, histone modifications, alterations in chromatin structure, and RNA-based mechanisms, play a pivotal role in controlling the gene expression of inflammatory pathways without altering the underlying DNA sequence.^85,86^ As aging progresses, epigenetic alterations occur sporadically in response to both exogenous and endogenous factors and are closely associated with healthspan and lifespan.^87–89^ Sex-specific differences have also been observed in genome-wide DNA methylation patterns and associations with several cardiometabolic traits and varying risks of CVD, including MI and stroke.^90,91^ These findings suggest that the epigenetic landscape might play a critical role in understanding disease phenotypes and tailoring sex-specific treatments. Epigenetic age is therefore emerging as a personalized and accurate predictor of biological age. It has been linked to numerous age-related diseases and mortality,^92^ while epigenetic age acceleration is associated with the presence of subclinical atherosclerosis, a process mediated by systemic inflammation.^93^ Epigenetic mechanisms can be modified by pharmacological agents, lifestyle interventions, or diet.^94–96^ Consequently, epigenetic clocks that track biological age may serve as valuable tools for interventions aimed at mitigating the effects of aging, especially if they can detect biological aging in young individuals who show no signs of disease.

#### 2.3.1 DNA methylation

DNA methylation is a dynamic process involving the addition of a methyl group by DNA methyltransferases (DNMTs) or its removal by ten-eleven translocation methyl-cytosine dioxygenases (TET), primarily targeting cytosine residues in CpG dinucleotide sites, leading generally to transcriptional repression. These enzymes are regulated by genetic and environmental factors and by age.^97,98^ DNA methylation influences the inflammatory response of circulating leukocytes, enhances the release of inflammatory cytokines, and promotes the progression of age-related CVDs.^99–102^ In the general population, over 10% of people older than 70 years harbor blood cell clones with loss-of-function mutations in epigenetic modifiers such as TET2 and DNMT3A.^103^ While 75% of CpG sites are typically methylated in mammalian cells, aging leads to deviations in global DNA methylation patterns. Global hypomethylation occurs alongside localized hypermethylation at specific loci, contributing to genomic instability.^104–107^ Atherosclerotic lesions in humans and preclinical models exhibit global DNA hypomethylation, while promoter regions of atheroprotective genes associated with endothelial and smooth muscle cell functions often show hypermethylation.^108–111^ Reduced DNA methylation has also been observed in the promoter region of TNFα,^112^ a potent inflammatory cytokine associated with vascular aging.^113,114^ Additionally, age-dependent DNA hypomethylation regulates interleukin (IL)-1β and IL-6 expression.^115,116^ Since the benefit of IL-1β-targeting therapies like canakinumab depends on the magnitude of the IL-6 response,^117,118^ DNA methylation levels may partly explain why patients with somatic variations in TET2 or DNMT3A face an increased risk of major adverse cardiovascular events.^119^

#### 2.3.2 Histone modifications and chromatin remodelling

Aging is associated with specific changes in histone levels and their numerous post-translational modifications, including ubiquitination, which alters chromatin structure and accessibility. This shift from tightly packed heterochromatin to loosely organized euchromatin leads to genomic instability, loss of silencing, and increased transcription of retrotransposons.^120–122^ Senescent cells accumulate senescence-associated heterochromatin foci, which silence cell cycle-related genes like E2F target genes.^123,124^ Changes in specific histones, such as decreased H3K9me3 levels and increased H4K20m33 and H3S10P levels, contribute to inflammageing, the gradually increasing activation of the immune system through aging.^125,126^ Sirtuins, a class of histone deacetylases (HDAC), regulate genes involved in NO signalling, oxidative stress, autophagy, and vascular aging through chromatin remodelling.^127^ HDAC inhibitors have been shown to significantly reduce TNF-α-stimulated VCAM-1 expression.^128^ Similarly, HDAC9 is linked to increased inflammation in advanced plaques, CAD, and ischaemic stroke.^129–131^ HDAC9-deficient mice exhibit reduced atherosclerotic lesions, while macrophage-specific HDAC9 deficiency upregulates histone H3 and H4 acetylation and increases ABCA1 and PPARγ levels, preventing cholesterol efflux.^132^

#### 2.3.3 RNA-based mechanisms

Non-coding RNA profiles, including microRNAs (miRNAs), long non-coding RNAs (lncRNAs), and circular RNAs (circRNAs), are profoundly affected by aging and are associated with all-cause mortality and age-related traits.^133–136^ These non-coding RNAs serve as critical regulators of multiple biological processes related to aging.^86,137^ Several miRNAs, such as miR-21, miR-146a, miR-155, miR-126, and miR-3a, are implicated in inflammageing.^137^ Increased levels of miR-34 and reduced expression of its target gene, SIRT1, have been identified in replicative-senescent human ECs, replicative-senescent human aortic smooth muscle cells, and aged mouse aortas.^138,139^ In humans, miR-34 is associated with aortic stiffness, a surrogate marker of arterial aging, and the presence of CAD.^140^ Notably, leukocyte-specific deletion of miR-34 mitigates atherosclerotic plaque development and enhances Sirt1 expression in an atherosclerosis mouse model.^140^ The lncRNA BACE1-AS has been shown to enhance BACE1 mRNA stability, promoting Aβ formation.^141,142^ This lncRNA is associated with accelerated vascular aging and the presence, extent, and incidence of atherosclerosis in humans.^143^ Although few studies directly link post-transcriptional regulation to inflammageing, accumulating evidence highlights the critical roles of RNA-binding proteins and RNA modifications in age-related diseases.^144–147^ The contribution of these RNA metabolism regulatory processes to inflammageing is poised to become a central focus of scientific research in the years ahead.

### 2.4 Endothelial dysfunction

Aging significantly impacts the vascular endothelium, the monolayer of ECs lining arteries, veins, and capillaries.^148^ ECs serve as gatekeepers, regulating the movement of molecules, nutrients, and immune cells between blood and tissues. In their quiescent state, ECs express factors that prevent leukocyte adhesion, platelet activation, and oxidative stress. However, aging leads to a progressive decline in endothelial function, shifting the endothelium towards a proinflammatory, vasoconstrictive, and prothrombotic state, ultimately increasing the risk of CVDs.^148^

A key factor in endothelial dysfunction is the reduced availability of NO, a vasodilator that regulates vascular tone and inhibits platelet aggregation. The decline in NO bioavailability reduces the ability of blood vessels to expand and contract, contributing to elevated blood pressure.^149,150^ The mechanisms underlying age-related endothelial dysfunction also include increased oxidative and nitrosative stress, cellular senescence, mitochondrial dysfunction, and impaired angiogenesis.^148,151–153^

Alongside SIRT1’s aforementioned role in RNA-based mechanisms, the sirtuin family members SIRT1 and SIRT3 have been implicated as important players tying together endothelial dysfunction, oxidative stress, mitochondrial dysfunction, and cellular senescence in age-associated CVD. Using novel techniques, it was recently shown that chronic, targeted delivery of the phenolic antioxidant compound esculetin to the mitochondria of human aortic ECs resulted in improved mitochondrial respiration and delayed senescence-like features through SIRT1 activation.^154^ Furthermore, chronic treatment with this targeted therapy alleviated age-associated atherosclerosis in *Apoe*^−/−^ mice. In aortic ECs, a reported contributing mechanism of esculetin’s beneficial effects is enhanced mitochondrial biogenesis via the AMPKα-SIRT3 axis but crucially, targeted delivery of esculetin to the mitochondria is required for its *in vivo* beneficial effect.^155^ Altogether, these studies highlight SIRT1 and SIRT3 as common players within interconnecting processes of CVD and aging, whilst also emphasizing the power of targeted, mechanistically-informed interventions like antioxidants.

With aging, ECs show increased expression of adhesion molecules promoting leukocyte recruitment to the arterial wall, a critical step in atherosclerosis development.^156,157^ Age-related alterations in cytokine levels, such as IL-6 and TNF-α, further drive inflammation and endothelial activation.^158^ Endothelial dysfunction is also linked to metabolic disorders such as diabetes and obesity,^159,160^ which worsen insulin resistance and increase the risk of vascular complications.^161^ The chronic inflammation associated with impaired endothelial function contributes to metabolic imbalances, providing a fertile ground for the development of age-related diseases.^162,163^

Structural changes in the arterial wall are also observed with aging, including luminal enlargement, intima and media thickening, and medial calcification. Smooth muscle cells undergo phenotypic switching, transforming into a synthetic, osteogenic, and pro-inflammatory phenotype.^149,164^ This shift alters the extracellular matrix (ECM) composition, increasing collagen and reducing elastin.^165^ Additionally, aged endothelium shows increased permeability,^166,167^ allowing infiltration of immune cells that produce ECM-degrading enzymes, such as matrix metalloproteinases.^168^ Medial arterial calcification leads to the precipitation of hydroxyapatite crystals in the arterial wall,^169^ further contributing to arterial stiffness, an independent predictor of incident CVD and all-cause mortality.^170^

In large arteries, endothelial dysfunction, combined with vascular wall remodelling and calcification, promotes atherosclerosis, a chronic inflammatory disease.^4,171,172^ Atherosclerotic lesions typically develop in areas of disturbed blood flow, damaging vascular ECs and triggering inflammation. Compromised endothelial integrity facilitates the accumulation of oxidized low-density lipoprotein particles, leading to monocyte differentiation into macrophages and foam cell formation, contributing to the plaque formation. This may lead to increased vascular resistance and platelet aggregation, raising the risk for hypertension, thrombosis, and acute cardiovascular events, such as MI and stroke.^4,171,172^ The relationship between endothelial dysfunction and atherosclerosis appears bidirectional, indicating that these pathological mechanisms exacerbate each other.^173,174^ Although aging-associated processes, such as endothelial dysfunction and media remodelling, equally affect veins and arteries, their effects in the two are dramatically different because of the haemodynamic differences. Indeed, human veins do not develop atherosclerosis, but the slower blood flow predisposes to thrombosis, especially in the lower limbs.^175,176^ In microvascular beds, aging impairs endothelial vasodilation, endothelial permeability, and reduces capillary density, to compromise the arterial myogenic tone, a mechanism of autoregulation, to maintain a relatively constant blood flow in the capillary bed.^177^ Aging-related changes in this process explain why older adults are more prone to hypertension-related complications, such as chronic kidney disease.

Understanding the complex interplay of mechanisms underlying endothelial dysfunction in aging offers promising insights into interventions that may slow or even reverse these processes. Regular physical exercise has been shown to improve NO production and maintain vascular elasticity.^178^ Dietary interventions, like the Mediterranean diet, can mitigate oxidative stress and inflammation.^179^ Pharmacological strategies, including cholesterol-lowering and blood pressure-lowering drugs, can further improve endothelial function.^180,181^ Similarly, KCa channel activator improved endothelium-dependent vasodilation, and prevented the aging-associated declines in cardiac ejection fraction.^182^ Senolytics, which remove senescent ECs *in vitro*, improve cardiac and endothelial function in aged mice.^183,184^ By targeting the cellular and molecular mechanisms driving endothelial aging, these interventions may delay or even reverse vascular dysfunction, ultimately reducing the burden of age-related CVD.

### 2.5 Oxidative stress

Oxidative stress, characterized by an imbalance between the production of ROS and the antioxidant systems that detoxify them, plays a central role in the accelerated progression of cardiovascular aging.^185^ This imbalance is particularly detrimental to the vascular endothelium, where excessive ROS generation impairs NO bioavailability, promotes inflammation, and accelerates endothelial dysfunction. Mitochondria, the primary source of ROS in cardiovascular cells, become progressively dysfunctional with age, contributing to a vicious cycle of oxidative damage and cellular senescence.^186,187^ Mitochondrial ROS not only damage DNA, proteins, and lipids but also activate redox-sensitive signalling pathways, further exacerbating vascular inflammation and apoptosis.^188^ In ECs, this oxidative burden reduces angiogenic capacity, impairs vasodilation, and increases vascular stiffness, which are precursors to age-related CVDs.^189^

The oxidative stress theory of aging, first proposed by Denham Harman in the 1950s, posits that accumulated ROS generated during normal aerobic metabolism cause damage to proteins, lipids, and DNA, thereby accelerating the aging process.^190^ Experimental evidence from genetically modified mouse models with altered expression of superoxide dismutase (Sod1 or Sod2), two key antioxidant enzymes, has provided compelling support for this theory. Sod1 knockout (KO) mice, lacking cytosolic superoxide dismutase, display elevated oxidative stress and an accelerated aging phenotype, characterized by muscle atrophy, weakness, and a 30% reduction in lifespan.^191–193^ Knockout of mitochondrial superoxide dismutase (Sod2) results in neonatal lethality due to dilated cardiomyopathy, supporting the essential role of mitochondrial ROS detoxification in cardiac development and survival.^194^ Heterozygous Sod2+/− mice, which survive into adulthood, exhibit age-dependent endothelial dysfunction, increased mitochondrial oxidative stress, and DNA strand breaks, further linking oxidative stress to vascular aging.^195^ Similarly, deficiency of the antioxidant enzyme glutathione peroxidase-1 in aged mice exacerbates vascular inflammation, characterized by monocyte and macrophage infiltration, oxidative DNA damage, and impaired endothelial function.^196^ In humans, patients with CAD and low red-cell GPx-1 activity are independently at higher risk of cardiovascular events,^197,198^ further highlighting the importance of endogenous antioxidant systems in maintaining vascular integrity during aging.

Importantly, oxidative damage extends beyond local vascular effects, contributing to systemic aging through genomic instability. Levels of oxidized DNA bases such as 8-oxo-2'-deoxyguanosine (8-oxo-dG) have been shown to inversely correlate with lifespan across species,^199^ suggesting that the efficiency of DNA repair and antioxidant defences may critically determine species-specific aging rates. The relevance of oxidative stress in premature vascular aging is further supported by studies of Hutchinson–Gilford progeria syndrome (HGPS), a genetic disorder characterized by accelerated aging. ECs derived from HGPS patients exhibit premature senescence, including telomere shortening, increased ROS production, and impaired angiogenic function.^200–202^ In hypertensive heart disease, oxidative stress accelerates telomere shortening, and elevated markers of telomeric damage serve as strong predictors of HF progression,^203^ confirming that ROS-induced genomic instability may mechanistically link molecular aging to clinical cardiovascular outcomes.

Oxidative stress also interacts with lifestyle and environmental factors that modulate cardiovascular aging.^204^ Interventions that reduce oxidative load, such as antioxidant-rich diets, regular physical activity, and pharmacological agents, have shown beneficial effects in both preclinical models and clinical studies. Empagliflozin, a sodium-glucose cotransporter 2 (SGLT2) inhibitor, has been shown to improve endothelial function and reduce mitochondrial oxidative stress in frail hypertensive and diabetic patients, highlighting a potential therapeutic strategy for mitigating vascular aging.^205^ Angiotensin II–induced upregulation of SGLT1 and SGLT2 promotes endothelial senescence and dysfunction via oxidative stress pathways, an effects that can be reversed by gliflozins treatment.^206^ Metformin, a widely used antidiabetic drug, has also been shown to extend healthspan by reducing oxidative stress, improving endothelial function, and modulating metabolic pathways.^207^ Taurine, a sulfur-containing amino acid with antioxidant properties, has been associated with improved endothelial function and reduced oxidative stress in aging models, suggesting its potential as a dietary supplement to counteract cardiovascular aging.^208^ Although the direct link to human aging remains to be fully clarified, these findings suggest that targeting oxidative stress pathways may offer benefits against age-related vascular decline.

While the role of oxidative stress in aging is multifaceted, mounting evidence indicates that it acts not only as a marker of biological aging but also as a driver of pathological cardiovascular changes. Thus, targeting oxidative mechanisms presents a promising strategy to delay the onset of CVDs and extend healthspan. However, it is essential to distinguish between physiological ROS signalling, which is crucial for normal vascular tone, immune defence, and cellular adaptation, and pathological oxidative stress, which overwhelms compensatory mechanisms. Future research must therefore focus on refining therapeutic approaches that restore redox balance without impairing vital cellular signalling pathways.

### 2.6 Inflammation and cardiovascular aging

Inflammageing refers to the increasing activation of the immune system through repeated antigenic stimulation through life.^209^ The aged immune system is simultaneously less effective at preventing and clearing infections^210^ and overactivated with higher circulating levels of cytokines and higher incidences of some autoimmune diseases, such as giant cell arteritis.^211–213^ By helping to unravel this seeming paradox, inflammageing provides insights into many CVDs of aging.

With aging, the composition of the immune cell repertoire changes. Lymphoid-biased haematopoietic stem cells (HSCs) persist in smaller numbers than myeloid-biased HSCs, leading to a myeloid shift in circulating immune cells.^214^ This phenomenon can be easily observed in the blood count of older individuals as a higher neutrophil-to-lymphocyte ratio (NLR), which correlates with both chronological age and markers of biological age, such as reduced grip strength.^215^ NLR also predicts cardiovascular outcomes, including cardiovascular mortality, in a range of clinical situations, suggesting this myeloid shift may be actively involved in CVD.^216,217^ MR analysis of a UK biobank cohort, however, has not found evidence for a causal link between NLR and CAD or MI.^218^

The causative association between systemic inflammation and CVD is well established, through both observational studies and clinical trials. C-reactive protein (CRP) levels predict long-term adverse cardiovascular outcomes similarly well, or better, than cholesterol levels,^219^ and inflammatory diseases such as rheumatoid arthritis and systemic lupus erythematosus are robustly associated with increased CVD risk.^220^ Systemic inflammation is also common in patients with atherosclerotic CVD (ASCVD), with a large study in Sweden finding 60% of patients with ASCVD have a CRP of 2 mg/L or higher. The inflammatory hypothesis of atherothrombosis was proven with the Canakinumab Anti-Inflammatory Thrombosis Outcome Study, in which the IL-1β inhibitor canakinumab was superior to placebo with regards to cardiovascular events in the secondary prevention setting—the first time an anti-inflammatory medication has been proven effective in improving cardiovascular outcomes. Later, trials of colchicine, another anti-inflammatory treatment, have further cemented the finding that changes in the immune system have a causative relationship with cardiovascular outcomes.^221–223^

Mitochondrial function, as an upstream regulator of inflammation, also represents a promising target for immunomodulatory therapies in CVD. In a randomized clinical trial of 90 post-MI patients aged over 65, twice-daily treatment with the mitochondrial telomerase activator TA-65 for 12–15 months resulted in reduced circulating inflammatory markers and increased numbers of adaptive immune cells compared with placebo.^224^ Patients receiving TA-65 also experienced 30% fewer adverse events, suggesting a therapeutic advantage over conventional anti-inflammatory agents, which are often limited by systemic side effects.

In summary, the aging immune system changes substantially, both through a myeloid shift of immune cells and cellular senescence, with SASP-induced inflammation. Inflammation is concretely associated with CV disease, and this inflammation of aging is a key mechanism for adverse CV outcomes in the elderly.

## 3. Complications associated with cardiovascular ageing

**Figure 2:**
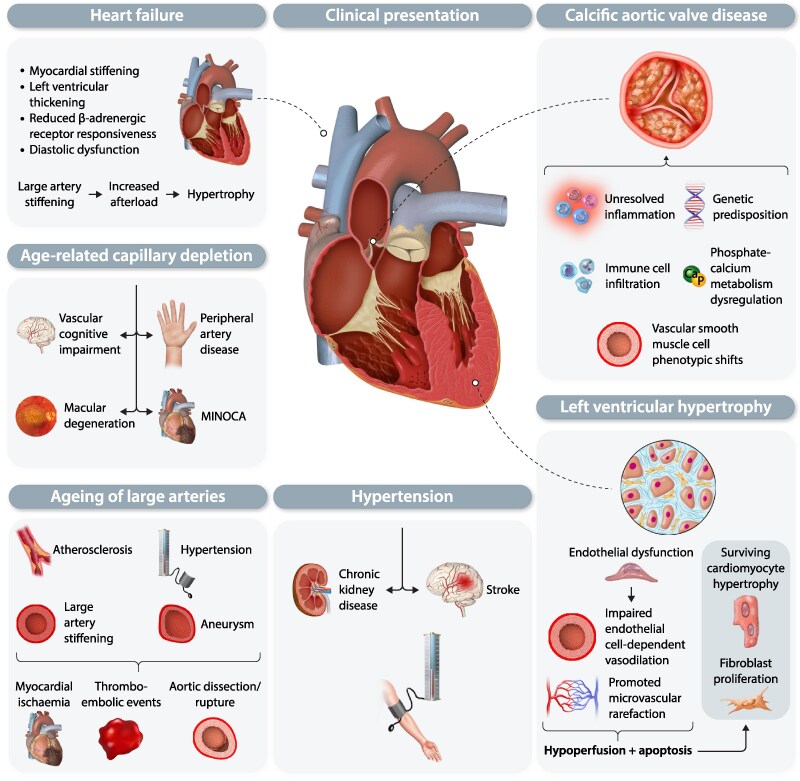
A summary of age-related complications in the cardiovascular system. MINOCA, myocardial infarction with non-obstructive coronary arteries.

Cardiovascular aging has a wide range of clinical consequences, and most acquired CVDs are linked to aging processes (summarized in *Figure 2*). Aging of large arteries is linked to increased arterial stiffness, hypertension, atherosclerosis, and aneurysm formation—this may result in myocardial ischaemia, thromboembolic events, and spontaneous dissection or rupture of the aorta, all of which carry a risk of fatal consequences.^4^ At the core of most aging-related CVDs lies the progressive dysfunction of the endothelium, which impacts arterioles and the microcirculation by impairing EC-dependent vasodilation^225^ and promoting microvascular rarefaction.^226^ The resulting myocardial hypoperfusion contributes to cardiomyocyte apoptosis and necrosis, which in turn accelerates hypertrophy of surviving cardiomyocytes and stimulates fibroblast proliferation, leading to further left ventricular (LV) hypertrophy.^227^

The loss of arterial myogenic tone^228^ in response to increased intraluminal pressure in older individuals heightens the risk of hypertension-related complications, including chronic kidney disease and stroke.^229^ Additionally, age-related capillary depletion is linked to vascular cognitive impairment,^228^ peripheral artery disease,^230^ and macular degeneration.^231,232^ In the myocardium, the relatively stable capillary network becomes dysfunctional, increasing the risk of MI with nonobstructive coronary arteries.^233^

Among structural heart diseases, calcific aortic valve disease (CAVD) with haemodynamically significant aortic stenosis is particularly prevalent in older individuals.^234^ Although its pathophysiology remains largely unclear, CAVD shares features with arterial stiffness and atherosclerosis,^235^ including genetic predisposition, immune cell infiltration, unresolved inflammation, vascular smooth muscle cell phenotypic shifts, and phosphate-calcium metabolism dysregulation.^236^

HF is a leading cause of morbidity in aging populations,^237^ characterized by myocardial stiffening, LV thickening, and reduced β-adrenergic receptor responsiveness.^238–241^ Large-artery stiffening increases LV afterload, leading to compensatory LV hypertrophy, which in turn raises myocardial oxygen demand.^235,242^ Although some studies suggest that aging reduces systolic contractility,^243^ diastolic dysfunction is more prevalent, as evidenced by the predominance of HF with preserved ejection fraction (HFpEF) over HF with reduced ejection fraction (HFrEF) in older adults.^244^ HFpEF, often secondary to ischaemic insults, is a key contributor to cardiovascular mortality, with diastolic dysfunction emerging as a hallmark of myocardial aging.^245^

The brain vasculature plays a critical role in maintaining cerebral homeostasis, by delivering oxygen and nutrients and removing waste products.^246^ Aging of the cerebrovascular system is associated with reduced elasticity of blood vessels, endothelial dysfunction, and decreased perfusion capacity.^247^ These age-associated vascular alterations not only impair nutrient and oxygen delivery but also compromise the blood–brain barrier, increasing susceptibility to neuroinflammation and neurodegeneration.^247^ Indeed, microvascular impairment and inflammation promote vascular cognitive impairment.^228,248,249^ Consequently, compromised cerebral blood flow, microvascular integrity, and vascular remodelling processes have been increasingly recognised as central contributors to age-related neurodegenerative conditions, particularly vascular dementia and Alzheimer’s disease.^250,251^ These conditions are particularly prevalent in the aging population and are frequently associated with cardiovascular risk factors such as hypertension, atherosclerosis, diabetes mellitus, obesity, and hyperlipidemia.^252–256^ Reciprocally, the presence of established cardiovascular risk factors predicts faster cognitive decline.^257^ As such, cognitive impairment is tightly connected to cardiovascular aging in a likely bidirectional manner.^258^ Importantly, these conditions do not act in isolation but interact synergistically with genetic predispositions and lifestyle factors to influence cognitive trajectories in aging populations.^259^ Given these multifaceted interactions, monitoring and managing vascular health, through lifestyle intervention, pharmacological treatment of cardiovascular risks, and early imaging biomarkers, may offer promising avenues for preventing or delaying the onset of vascular cognitive impairment.

## 4. Measuring cardiovascular ageing

### 4.1 Clinical biomarkers

Hypertension is among the strongest predictors of incident CVD, and is closely tied to aging.^260^ Broadly, hypertension can be classified as either isolated diastolic (IDH), isolated-systolic (ISH), or systolic-diastolic (SDH), depending which of the systolic and diastolic blood pressure (BP) are above the reference limit. Data from the National Health and Nutrition Examination Survey (NHANES), a large population study in the United States, demonstrate that ISH and SDH predict an increased risk of cardiovascular events, while IDH does not.^261,262^ Older patients in this study had a remarkably different profile of hypertension. The prevalence of IDH decreased steadily from 39.2% in patients under 40 years old to 0.2% in those over 80, while the prevalence of ISH peaks in the 7th and 8th decades, largely due to arterial stiffness.^263^ It is well established that BP then starts to fall in the very old, and that lower BP in the elderly predicts all-cause mortality and even cardiovascular events.^264–266^ This makes BP a complex marker of aging in the cardiovascular system, with the trend of a person’s BP over many years giving important prognostic information.

Multiple genome-wide association (GWA) studies have sought a heritable basis for extreme longevity, and the SNP rs429358 [apolipoprotein E (ApoE) ε4] is consistently associated with lower odds of longevity, while other ApoE variants (ε2 and ε3) are associated with greater odds.^267,268^ ApoE is the major carrying molecule for lipids, and its consistent association with longevity highlights the major impact of atherosclerotic disease on lifespan. ApoE ε4 may also hold promise as a novel biomarker, particularly for Alzheimer’s dementia.^269,270^

Markers of systemic inflammation, including C-reactive protein (CRP), IL-6, and IL-1β increase steadily with age. These markers are also robustly associated with CVD. CRP is associated with arterial stiffness^271,272^ and carotid calcification,^273^ although interestingly not with coronary artery calcium (CAC) score.^274^ IL-6 and IL-1β, which are upstream of CRP in the same axis, also associate with subclinical and clinical atherosclerosis.^275^ Interestingly, MR studies show that genetically lower CRP does not reduce CVD outcomes, while lower IL-6 does.^276^ Conversely, polymorphisms which increase the level of the IL-1 receptor antagonist, IL-1R, and so decrease the activity of IL-1, are associated with higher incidence of coronary heart disease.^277^ These data suggest that IL-6 may be the key signalling molecule in this pathway, and uniquely amenable to treatment. Clinically, CRP is currently the only inflammatory biomarker for CVD recommended by international guidelines, with the American Heart Association (AHA) recommending its measurement for more detailed assessment of cardiovascular risk.^278^

### 4.2 The blood-based peptide amyloid-beta 1–40

**Figure 3:**
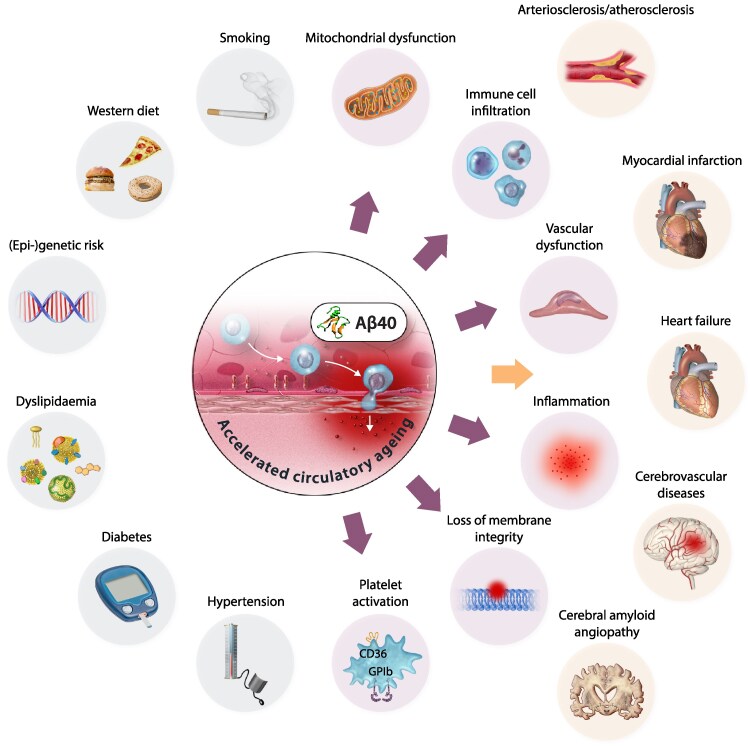
Central role of amyloid-β 1-40 in vascular aging and its contribution to both cardiovascular and neurovascular diseases. Lifestyle and genetics factors, such as a Western diet, smoking, genetic predisposition, dyslipidemia, diabetes mellitus, and hypertension contribute to accelerated cardiovascular aging. The subsequent elevated circulating levels of Amyloid-β 1-40 leads mitochondrial dysfunction, immune cell infiltration, vascular dysfunction, inflammation, loss of membrane integrity, and platelet activation, ultimately promoting arteriosclerosis, myocardial infarction, heart failure, cerebrovascular diseases, and cerebral amyloid angiopathy. Amyloid-β 1-40 as a central driver of the interplay between metabolic, inflammatory, and vascular pathways emerges as a valuable biomarker of cardiovascular aging. Figure created with Biorender: https://BioRender.com/yogrvis.

A new marker of biological age holding great potential is the amyloid-beta 1-40 (Aβ40) blood-based peptide, which is linked to several CVDs (*Figure 3*). Aβ is a proteolytic fragment of the amyloid precursor protein (APP), known for its involvement in Alzheimer's disease.^142^ APP is produced in neurons, platelets, cardiomyocytes, and all vascular cells.^141,142^ β-secretase (BACE1) is involved in APP cleavage, and further cleavage by γ-secretases generates peptides of length 40 (Aβ40), which is found in vascular lesions, and 42 (Aβ42), which is associated with brain lesions in Alzheimer’s disease. The BACE1 antisense transcript (BACE1-AS), a conserved long noncoding RNA, has been found to enhance BACE1 mRNA stability and thus promote Aβ formation.^279,280^ Several factors, including inflammation, renal dysfunction, or ischaemia, increase circulating levels and subsequent tissue deposition of Aβ by augmenting its production and processing or by decreasing Aβ clearance.^141,142^ Under normal conditions, equilibrium exists between Aβ production and removal. Deregulation of this balance may lead to CVD-associated accumulation of Aβ in the blood, vessels, and heart.^141,142^ Increased APP processing and Aβ production may be directly linked to endothelial dysfunction in cerebral and peripheral blood vessels. Aβ peptides at high concentrations are toxic to brain and peripheral ECs, causing cellular damage, enhanced vasoconstriction, and impairment of endothelium-dependent relaxation, thereby promoting atherosclerosis, an age-related disease.^281^ Additionally, APP and Aβ have been detected in human carotid plaques and atherosclerotic aortas.^282^ Overexpression of APP accelerates atherosclerosis,^283^ while APP deletion partially protects against the development of aortic atherosclerosis in ApoE^−/−^ mice.^284^ Clinical and experimental evidence indicates that Aβ may play a crucial role not only in the brain but also in the general vasculature. Amyloid deposits are found in the aortic walls of almost 100% of individuals over 50 years of age.^285^ Interestingly, the aortas of elderly individuals with either mild fatty streaks or advanced atherosclerotic lesions predominantly contain Aβ40 peptides.^286,287^ Furthermore, elevated circulating amyloid concentrations in obesity and diabetes promote vascular dysfunction.^288^ Although the source of elevated plasma Aβ40 levels in aged humans remains unknown, endothelial APP significantly contributes to blood Aβ levels, as shown in mice.^289^ Unexpectedly, it has been recently proposed that upregulation of APP in the vascular endothelium of aging mice may be an adaptive response designed to protect endothelial function.^290^ Altogether, these data suggest that while endothelial Aβ production is necessary for normal function, an excess could contribute to age-related arterial stiffening.

Increased plasma Aβ40 levels have been associated with subclinical cardiac disease, as indicated by elevated high-sensitivity cardiac troponin T, N-terminal pro-B-type natriuretic peptide, and lower LV ejection fraction.^291,292^ Additionally, plasma levels of Aβ40 are associated with declining cardiorespiratory fitness in patients without clinically overt CVD.^291^ Elevated circulating Aβ40 levels have also been observed in patients with hypertension, diabetes mellitus, and dyslipidemia.^293^ In a prospective study of healthy young to middle-aged adults, changes in plasma Aβ40 levels were found to independently predict changes in aortic stiffness, a surrogate marker of vascular aging.^291^ In patient at risk for ASCVD, Aβ40 is associated with all-cause mortality partly mediated through renal dysfunction.^294^ Aβ40 levels are associated with the presence, extent, and progression of carotid atherosclerosis in postmenopausal women^295^ and are linked to plaque composition and burden in patients without clinically overt atherosclerotic CVD.^296^ Circulating Aβ40 provides incremental prognostic value and enhances risk stratification in patients with stable CAD and non-ST elevation acute coronary syndrome for predicting adverse cardiovascular events.^297,298^ It is also independently associated with mortality in HF patients, possibly due to worsening cardiac function.^292,297,298^ Its prognostic significance has largely been demonstrated through retrospectively analyzed prospective studies. Implementing bedside Aβ40 measurement tests in clinical trials could facilitate the establishment of baseline levels and thresholds for predicting adverse outcomes across various age groups. Notably, numerous effective anti-aging strategies have been found to enhance Aβ40 metabolism, highlighting its pivotal role in the aging process.^141^

### 4.3 CHIP

Clonal haematopoiesis (CH) describes the process when haematopoietic stem and progenitor cells (HSPCs) acquire mutations in genes known to be associated with haematological malignancy but in the absence of an overt blood disorder. These mutations are passed on to progeny cells, resulting in clones of mutant cells that are detectable in the peripheral circulation. CH is defined when the variant allele frequency (VAF) is ≥1% and has been associated with several age-related CVDs. These include atherosclerosis, ischaemic heart disease, MI, HF, as well as outcomes from CVD, including death.^299–303^

CH is an age-related phenomenon as mutations are acquired over time, affecting ∼10% of those over the age of 65 years in population studies.^304^ Age is also strongly associated with the presence of CH in most reported disease cohorts, as well as being a predictor of accelerated clonal growth.^305^ The most frequently identified mutations are in *DNMT3A* and *TET2* genes, genes which encode enzymes responsible for methylating and demethylating DNA CpG sites, respectively. *DNMT3A*- and *TET2*-CH confers abnormal function to mature blood cells, leading to predominantly pro-inflammatory effects.^300,301^

Other than age, clonal growth is also promoted by several established, cardio-metabolic risk factors, including atherosclerosis,^306^ obesity,^307,308^ and smoking.^309^ It is also associated with metabolic dysfunction, specifically low HDL cholesterol levels, which was not observed in patients who underwent bariatric surgery.^308^ CH progression has also been potentiated in a mouse model of obesity, which was partially recovered by anti-inflammatory treatment.^307^ Similarly, atherosclerosis has been shown to exacerbate stem cell division and clonal expansion,^306^ while smoking was shown to be a causal risk factor for CH in MR studies performed in the UK Biobank.^309^ Interestingly, this study also identified longer LTL as a causal risk factor for CH. Given the association between telomere shortening and cellular senescence,^310^ this suggests a complex relationship between cellular aging and clonal expansion.

It is therefore not known to what extent CH contributes to or is a consequence of aging. The first human evidence of causality has emerged from the Progression of Early Subclinical Atherosclerosis (PESA) study.^311^ Based on longitudinal CH assessment and serial vascular imaging, this study suggested that CH has a unidirectional, causative association with the development of atherosclerosis. Specifically, having a mutation related to CH, especially at higher VAF, was associated with an increased risk of developing *de novo* femoral atherosclerosis over 6 years, but the presence or severity of atherosclerosis did not influence clonal expansion over the same period. The causal effect of CH is supported by several animal studies, particularly those investigating *TET2* mutations,^312^ but has not yet been tested in clinical perturbation studies.

Therefore, while CH is inherently linked with aging, its association with age-related CVD suggests that it could both contribute to and result from systemic aging processes.

### 4.4 Imaging techniques

An arsenal of imaging techniques is available to help characterize cardiovascular aging. Echocardiography is the most widely used and accessible test of ventricular function and is vital in the diagnosis of HF with both reduced and preserved ejection fraction (HFrEF and HFpEF, respectively). HFpEF, especially, is largely a disease of aging, with ventricular stiffness in diastole commonly identified in older echo subjects.^313^ Measures of diastolic dysfunction, such as a dilated left atrium (LA), reduced E/A ratio and increased E/e’ ratio are useful measures of cardiovascular aging even in the absence of a diagnosis of HFpEF.^314^ Atria abnormalities such as dilatation may represent ‘atrial cardiopathy’,^315^ which is a strong predictor of both incident atrial fibrillation (AF) and stroke. However, clinical trials in patients with atrial cardiopathy, but not AF, have not yet yielded strong evidence for anticoagulant use.^316^

Measured with B-mode ultrasound, the carotid artery intima-media thickness (CIMT) is a measure of subclinical atherosclerosis and is well studied as a risk-stratifying tool.^317^ Although a higher CIMT correlates with higher event rates, it remains unclear whether CIMT measurement in clinical practice improves outcomes, and it is therefore not recommended for this purpose in international guidelines.^318^ CIMT increases robustly with age, and this appears to influence its predictive power, with one retrospective study finding CIMT improved risk stratification for cardiovascular death only in younger patients.^319^ Each standard deviation increase in CIMT was associated with a 27% increased risk of cardiovascular death in the 35–44 years age group, but only a 14% increase in the 65–74 years group.^319^ CIMT may therefore be most valuable as an assessment of premature cardiovascular aging, losing value in those who are already old.

Coronary calcium deposition is a hallmark of atherosclerosis and cardiovascular aging,^320,321^ and CT imaging provides rapid, non-invasive estimation of the burden of calcium in coronary arteries—a CAC score.^322^ CAC increases with age, and can therefore be used to give an ‘estimated coronary age’.^323^ This gives vital information in assessing risk for atherosclerotic CVD but also gives a powerful way to relate this risk to a patient.

In comparison to CT and ultrasonography, cardiac MRI has higher spatial resolution and so produces the most detailed assessment of myocardial tissue structure,^324,325^ making it an attractive modality for multiparametric assessment of myocardial aging. Several studies have applied deep learning algorithms to the UK Biobank population, analysing genetic associations with markers of ventricular stiffness, diastolic dysfunction and aortic distensibility, among other MR evidence of aging.^326–328^ These studies have shown that aortic and left atrial dimensions appear particularly predictive of aging and contribute the most to a synthesized age-prediction model based on imaging parameters.

Together, these varied imaging techniques allow powerful assessment of myocardial aging and risk stratification in older individuals.

### 4.5 Vascular function assessment

Arterial stiffness and endothelial dysfunction are a fundamental mechanism of many CVDs of aging, including hypertension, atherosclerosis and thrombosis^329^ and both can be measured non-invasively.

The carotid-femoral pulse wave velocity (PVW) is the gold-standard non-invasive assessment of central arterial stiffness, with relatively simple and reproducible measurement and a large body of evidence supporting its association with CVD.^330–335^ PVW uses electrocardiography and tonometry to measure the delay for an arterial pulsation to arrive at the carotid and femoral arteries, with stiffer arteries transmitting the pulsation more quickly. Notably, PVW values increase substantially with age, even in patients without CVD and normal blood pressure, and increase even more sharply in older patients with hypertension.^330^ This suggests that arterial stiffness, as measured by PVW, is a usable surrogate marker for cardiovascular age.^336^

Coronary artery endothelial dysfunction in atherosclerosis was first demonstrated with paradoxical vasodilatation after injecting acetylcholine into diseases coronary arteries,^337^ but technique is invasive and difficult to perform. Non-invasive assessments of endothelial dysfunction have since been introduced, including flow-mediated dilatation (FMD), which broadly assess the microvasculature, and peripheral arterial tonometry (PAT), assessing the microvasculature.^338^

FMD, which measures endothelium-dependent vasodilation, involves visualizing the brachial artery with ultrasound and then occluding the artery distally, using a cuff inflated to supra-systolic pressure. The dilatation of the brachial artery is then measured using ultrasound, with less dilatation indicating endothelial dysfunction. Lower FMD is associated with the presence and progression of atherosclerosis,^339,340^ and the occurrence of cardiovascular events,^341–343^ but its lack of incremental predictive power over traditional risk factors has prevented its recommendation in guidelines.^338^

An alternative measure of endothelial dysfunction in the PAT technique, in which a finger probe measures changes in the pulse waveform before and after reactive hyperaemia, which is induced with temporary occlusion of arterial flow with an inflated cuff.^344^ This has the advantage of being much less operator-dependent than FMD measurement, although the body of evidence linking PAT measurements to CVD, while present, is less robust.^345,346^

## 5. Modelling cardiovascular ageing

Traditionally, statistical models have played a central role in estimating cardiovascular risk and the progression of cardiovascular aging. Among the most established models is the Framingham Risk Score, which estimates a 10-year risk of cardiovascular events based on key clinical variables such as age, blood pressure, cholesterol levels, smoking status, and the presence of diabetes.^347^ Additional risk models like SCORE (Systematic COronary Risk Evaluation)^347,348^ and QRISK^349^ have been developed to tailor cardiovascular risk prediction to specific population groups. These traditional models predominantly use linear and logistic regression techniques, which assume a relatively linear progression of cardiovascular aging and focus on a restricted set of modifiable and non-modifiable risk factors.

While instrumental in clinical risk stratification, these models present significant limitations. They often struggle to capture complex, non-linear interactions among diverse biological processes and are not designed to integrate high-dimensional data sources, such as genomics, metabolomics, or imaging modalities. To address these shortcomings, systems biology approaches have emerged, leveraging the integration of multi-omics data—including genomic, transcriptomic, and proteomic profiles—alongside clinical and environmental variables to build more comprehensive predictive models of cardiovascular aging.^350^ Network-based modelling further facilitates the identification of molecular pathways and biomarkers that underpin cardiovascular aging, providing a foundation for targeted therapeutic interventions.^351^

Recent findings underscore the utility of such integrative approaches. A study involving over 6200 middle-aged individuals demonstrated that organ-specific proteomic signatures are predictive of long-term risk for age-related diseases.^352^ Notably, the study found high organ specificity for chronic HF and dilated cardiomyopathy among participants exhibiting a pronounced heart-age gap.

In parallel, advances in machine learning (ML) and artificial intelligence (AI) have revolutionized the modelling of cardiovascular aging. In contrast to traditional regression-based methods, ML techniques can manage extensive, multidimensional datasets and detect complex non-linear relationships that influence cardiovascular aging. Various ML strategies—including ensemble models that integrate random forests, support vector machines, neural networks, and deep learning—have been successfully applied to large datasets such as the UK Biobank, comprising over 375 000 individuals, to enhance cardiovascular risk prediction.^353^ Notably, the incorporation of mental health questionnaire data into the ensemble algorithm significantly improved predictive accuracy, increasing CVD risk prediction from 71% to 85%.

Biological aging clocks, which estimate biological age and highlight the divergence from chronological age, may also be valuable tools in modelling cardiovascular aging and capturing individual aging dynamics.^354^ An effective clock would also be sensitive to interventions or drug effects. Current clocks are based on measures such as epigenetic changes, inflammatory markers, plasma proteomics, or metabolomics (*Table 1*). However, their low intercorrelation suggests they capture distinct facets of the aging process.

**Table 1:** Blood-based biological clocks from large or midsized studies that predict mortality and/or cardiovascular relevant endpoints (adapted from Liberale *et al*., 2025)^148^

Blood-based biological clocks	Description	Predictive value for lifespan and/or healthspan
Haematological aging clock^355^	Several deep learning-based biological age predictors trained on 20 population-specific blood biomarkers and cell counts.	Associated with all-cause mortality
DNAmAge^354^	DNAm age is a molecular readout reflecting intrinsic aging processes and is defined as the predicted biological age	Associated with increased risk for all-cause mortality and CV disease. Diet and lifestyle treatment leads to a decrease in DNAmAge.
GlycanAge^356^	A biological age test measuring chronic inflammation via blood-based glycan profiles on IgG antibodies, which correlate with chronological age	Associated with multiple diseases, among others CV disease and diabetes.
PhenoAge^356^	An epigenetic clock based on DNA methylation at CpG sites strongly correlated with chronological age	Associated with risk of cancer, Alzheimer’s disease, CHD.
Proteomic clocks^357^	Proteomic clocks use protein biomarkers as intermediate phenotypes closely linked to age-related diseases, offering potentially greater accuracy in assessing aging and pathology	May predict CV death
Metabolomic clock^358^	Metabolomic clocks assess metabolites and small molecules as key links between genotype and phenotype in aging and age-related disease	Associated with all-cause, CV, cancer- and infection-related mortality
iAGE (Inflammatory aging clock)^359^	A blood-based immune biomarker metric for chronic inflammation, used to predict aging phenotypes and understand vascular aging mechanisms	Associated with exceptional longevity in centenarians. Associated with multimorbidity, immunosenescence, frailty and CV aging.
IMM-AGE (Immune aging score)^360^	A high-dimensional immune aging trajectory that more accurately reflects immune status than chronological age.	Better performance in predicting mortality in older adults than the epigenetic clock.

In conclusion, the modelling of cardiovascular aging has progressed from conventional statistical models to sophisticated, AI-driven approaches and systems biology frameworks that offer deeper insights and improved predictive capability.

## 6. Future directions

The modelling of cardiovascular aging has progressed from conventional statistical models to AI-driven approaches and systems biology frameworks that offer deeper insights and improved predictive capability. By integrating multi-dimensional biomarkers, such as epigenetic clocks, proteomic profiles, and arterial stiffness measures, personalized models that can quantify biological age and capture individualized vascular aging trajectories will be a transformative frontier in preventive and precision medicine.^352,361,362^ Furthermore, continuous monitoring allowing dynamic risk assessment through wearable technology and digital health tools can enhance these models by providing real-time data on physical and chemical signals that reflect the health conditions of older adults.^363^ Sex-specific factors, including differential vascular biology, plaque vulnerability, and hormonal influences, must be systematically incorporated in these models to enhance predictive accuracy, particularly given the accelerated vascular stiffening observed in postmenopausal women and distinct CVD manifestations between sexes.^364–366^ Emerging insights into epigenetic, clonal haematopoiesis and neuro-cardiovascular axes further underscore the need to expand biomarker panels to better reflect systemic aging processes.^301,367,368^ In that view, Aβ40 holds great potential for predicting adverse outcomes across life.^141,142^ However, critical challenges persist, including the standardization of measurements, validation of biomarkers across diverse ethnic, socioeconomic, and gender-diverse populations, and the establishment of causal links through large longitudinal studies. Ethical and regulatory frameworks must also evolve to support the translation of predictive models into clinical practice, particularly for preventive gerotherapeutics. By bridging mechanistic insights with clinical innovation, personalized models hold promise not only for mitigating CVD burden but also for redefining healthy aging paradigms in our increasingly diverse and aging global population.
